# Protracted Hypocalcemia following 3.5 Parathyroidectomy in a Kidney Pancreas Recipient with a History of Robotic-Assisted Roux-en-Y Gastric Bypass

**DOI:** 10.1155/2018/2182083

**Published:** 2018-07-24

**Authors:** Hugo Bonatti, Naureen Iqbal, Catherine Kling, Willie Melvin, James Broome

**Affiliations:** ^1^Department of Surgery, Vanderbilt University Medical Center, Nashville, TN, USA; ^2^Meritus Surgical Specialists, Hagerstown, MD, USA; ^3^Geisinger, Wilkes-Barre, PA, USA; ^4^Surgery, University of Washington, Seattle, WA, USA; ^5^StoneCrest Medical Center Clinic, Smyrna, TN, USA; ^6^St. Thomas Endocrine Surgical Specialists, Nashville, TN, USA

## Abstract

**Background:**

Hypocalcemia is a frequent complication of parathyroidectomy for secondary/tertiary hyperparathyroidism. In patients with a history of prior Roux-en-Y gastric bypass (RYGBP), changes in nutritional absorption make management of hypocalcemia after parathyroidectomy difficult.

**Case Report:**

A 41-old-year morbidly obese female with c-peptide negative diabetes mellitus and renal failure had RYGBP. Following significant weight loss she underwent simultaneous pancreas-kidney transplantation. She had excellent transplant graft function but developed tertiary hyperparathyroidism with calciphylaxis. She underwent resection of 3.5 glands leaving a small, physiologic remnant remaining in situ at the left inferior position. She was discharged on postoperative day one in good condition, asymptomatic with serum calcium of 7.6 mg/dL and intact PTH of 12 pg/mL. The patient had to be readmitted on postoperative day #14 for severe hypocalcemia of 5.0 mg/dl and ionized calcium 2.4 mg/dl. She required intravenous calcium infusion to achieve calcium levels of >6.5 mg/dl. Long-term treatment includes 5 g of elemental oral calcium TID, vitamin D, and hydrochlorothiazide. She remains in the long term on high-dose medical therapy with normal serum calcium levels and PTH levels around 100 pg/mL.

**Discussion:**

Our patient's protracted hypocalcemia originates from a combination of 3.5 gland parathyroidectomy, altered intestinal anatomy post-RYGBP, and potentially her pancreas transplant causing additional metabolic derangement. Alternative bariatric procedures such as sleeve gastrectomy may be more suitable for patients with renal failure or organ transplants in whom adequate absorption of vitamins, minerals, and drugs such as immunosuppressants is essential.

## 1. Introduction

Pancreas transplantation (PTx) has evolved as a reliable therapy for insulin-dependent diabetes mellitus (IDDM) and most commonly it is performed as a simultaneous pancreas-kidney transplant (SPK) in the setting of diabetic nephropathy [1, 2]. Only a limited number of patients with non-IDDM have thus far undergone PTx if they were able to lose significant weight and still remain insulin-dependent [3–5]. Obesity has become one of the most common disorders worldwide, and morbid obesity is often a contraindication for kidney and pancreas transplant listing. As nonoperative measures are unsuccessful in the majority of obese individuals, bariatric surgery has gained importance in the therapy of morbid obesity [6]. Currently in the US, the Roux-en-Y gastric bypass (RYGBP) is most commonly performed; the role of adjustable gastric banding (AGB) is declining; and gastric sleeve resection has been gaining importance [7, 8]. One major complication of RYGBP is development of malabsorption of various nutrients, electrolytes, vitamins, and some drugs. Poor absorption of calcium can be of particular concern in patient subsets and may lead to secondary hyperparathyroidism [9–11].

One of the most common secondary complications of DM is renal failure, which commonly is associated with secondary hyperparathyroidism [12]. Although renal transplantation may reverse secondary hyperparathyroidism, commonly tertiary hyperparathyroidism has already developed and, therefore, removal of the parathyroid glands may be required. Most surgeons prefer 3.5 gland resection; however, also total parathyroidectomy with autotransplantation has been suggested [13]. Hypocalcemia is a common complication after parathyroidectomy and in severe cases is also referred to as hungry bone syndrome [14]; it seems to originate from a rapid drop in the PTH levels and may be more common in patients with secondary hyperparathyroidism. The profound hypocalcemia and hypophosphatemia may linger and require high-dose calcium and vitamin D supplementation. Administration of bisphosphonates has been suggested to prevent and/or treat the condition.

We herein report a 35-year-old woman with IDDM, who developed severe hypocalcemia after three-and-a-half-gland parathyroidectomy for tertiary hyperparathyroidism in the setting of being status post-RYGBP and SPK.

## 2. Case Report

A 35-year-old female was diagnosed with type I DM at the age of 9 years. During childhood her DM was poorly controlled and the patient gained significant weight. At the age of 25 years her weight was 105 kg with a body mass index (BMI) of 40 kg/m2 and her renal function started to deteriorate with progression to requiring hemodialysis by age 30. With development of renal failure, secondary hyperparathyroidism was noted. Due to her obesity, she was not eligible for a renal transplant or a SPK. At this point it was decided to offer her bariatric surgery, and, after extensive discussion, it was felt that a RYGBP was the best option for her in terms of weight loss. At the age of 32 years, she underwent uneventful robotic-assisted surgery; the stomach remnant was attached to the abdominal wall for potential future access.

Over the next 2 years she lost 60 kg and underwent SPK during which the donor duodenal segment was diverted to a bowel loop distal to her Roux loop implant site into the common channel. Induction immunosuppression with alemtuzumab was followed by maintenance with tacrolimus (trough levels 5-7 ng/mL), mycophenolate-mofetil (2 g daily), and a steroid taper. She was CMV seronegative and received a graft from a CMV positive donor and received standard prophylaxis with oral ganciclovir (GCV) for 100 days. Within 100 days posttransplant, she was readmitted to the hospital with acute CMV disease, which was successfully treated with intravenous ganciclovir.

Shortly after this episode the patient was found to have skin lesions on her right leg, which were diagnosed as calciphylaxis. Her serum calcium at that time was 14 mg/dl and the diagnosis of tertiary hyperparathyroidism was made. A three-and-a-half-gland resection together with subtotal thymectomy was done without any complications; the left lower parathyroid gland was the only normal appearing and half of it was preserved taking care that blood supply remained intact. Intraoperative parathyroid hormone levels dropped from >1500 to 150. Calcium serum levels within 24 hours postoperatively were 9 mg/dl with ionized calcium of 3.5 mg/dl. She was discharged in good condition within 24 hours postoperatively with daily calcium supplementation of 4.5 g/day divided into three doses.

During the following week her calcium levels started to drop and on day 10 postoperatively at an outside hospital serum calcium was found to be critically low at 5.5 mg/dl with an ionized fraction of 2.1 mg/dl. However, the patient had remained clinically symptom free. She was admitted for intravenous calcium replacement. Pushes of calcium were unable to appropriately raise her calcium levels and, therefore, a calcium drip (85 mg/h) was started. Her calcium levels came up to 7.1 mg/dl. Oral calcium dose was raised to 15 g/day and hydrochlorothiazide was started. The calcium drip was stopped and the patient was discharged home in good condition.

Intense calcium supplementation was continued. Gradually the patient's gastrointestinal tract started to adapt and after two years her calcium levels started to stabilize. She has not experienced any additional complications from her transplant or gastric bypass. She is currently alive with excellent function of both grafts, normal calcium levels, stable weight, and an excellent quality of life almost five years after her last surgery.

## 3. Discussion

We report a patient with a complex medical history and multiple surgical interventions involving bariatric, transplant, and endocrine surgeons. The patient benefited from each of these interventions and all were clearly indicated. However, better communication between the various involved teams may have resulted in an even better outcome and prevention of the severe hypocalcemia following her three-and-a-half-gland parathyroidectomy. She was cured from her type 1 and type 2 DM and since her successful SPK she is off any antidiabetic medication and is off dialysis and has regained an excellent quality of life having maintained a normal weight after bariatric surgery.

Development of obesity in patients with type I DM is rare and is associated with an excessive risk for secondary complications due to the superimposed peripheral insulin resistance [15]. Escalating amounts of exogenous insulin may be required in these patients in order to maintain acceptable blood glucose control. In combination with other comorbidities, development of secondary complications including renal failure is common such as in our patient. Once on dialysis, renal transplant was discussed but her obesity was considered a contraindication. Therefore, bariatric surgery was suggested and RYGBP seemed to be the most promising intervention to rapidly achieve the necessary weight loss.

Hypocalcemia is more common after RYGBP than after AGB or gastric sleeve resection as calcium absorption takes place mainly in the proximal gastrointestinal tract which is excluded in patients with RYGBP [16]. However, sleeve gastrectomy also may cause secondary metabolic complications with low calcium and elevated PTH levels [17]. Successful AGB has been reported in a SPK recipient who developed significant weight gain [18] and another SPK recipient underwent placement of a gastric pacemaker for gastroparesis associated with significant weight gain [19]. Limited data on sleeve gastrectomy in PTx are available but the procedure seems promising and appealing in this patient population [20–22]. Small series of patients with NIDDM undergoing PTx have been published; the treatment is reserved for patients who have lost significant weight and remain insulin-dependent [23].

In our patient, management of hypocalcemia was challenging [24]. Hungry bone syndrome may have been an important component leading to the very low serum calcium levels [14]. It is unclear if a RYGBP or a SPK transplant may exacerbate hungry bone syndrome. Even with 15 g of oral calcium and administration of vitamin D, the patient had difficulties to maintain normal calcium levels due to limited calcium absorption. Vitamin D levels were kept above 50 ng/ml throughout the postoperative course. Palal et al. reported on life-threatening hypocalcemia after parathyroidectomy in a renal failure patient with previous RYGBP [24]. Hydrochlorothiazide increases calcium reabsorption and decreases renal calcium loss; however, use of diuretics in SPK recipients is controversial.

If the patient would not have been able to maintain adequate calcium levels in the long term, placing a gastrostomy tube into the stomach remnant under fluoroscopic guidance may have been an option. Calcium could be delivered directly into her proximal gastrointestinal tract, with improved absorption and resolution of hypocalcemia. Recently, recombinant parathyroid hormone has been introduced in clinical practice [25] for refractory cases of hypoparathyroidism but is reluctantly used in patients like ours. In fact PTH levels recovered in our patient and ultimately she was able to adjust to her unique metabolic challenge with high-dose oral calcium and vitamin D administration. This complication of severe hypocalcemia after parathyroidectomy should be considered in patients with a history of RYGBP [26, 27].
